# 65/m – Eingeschränkte Lebensqualität und Mobilität bei Schmerzen in Beinen und Rücken: Vorbereitung auf die Facharztprüfung: Fall 61

**DOI:** 10.1007/s00132-021-04076-x

**Published:** 2021-02-24

**Authors:** D. Sauer

**Affiliations:** 1 Wirbelsäulenzentrum, Schön Klinik München Harlaching, Harlachinger Str. 51, 81547 München, Deutschland; 2 Paracelsus Medical University, Salzburg, Österreich

**Keywords:** Claudicatio-spinalis-Symptomatik, Gehstreckenlimitierung, Nervenkompression, Redundante Nervenwurzeln, Schizas-Klassifikation

## Prüfungssimulation

### Fallschilderung

Der 65-jährige Herr H. – langjähriger Raucher – stellt sich in Begleitung seiner Ehefrau in Ihrer orthopädischen Sprechstunde vor. Er berichtet über eine beginnende Einschränkung der Lebensqualität und Mobilität aufgrund von Schmerzen bei Belastung in den unteren Extremitäten und dem Rücken. So ginge es nicht mehr weiter, er sei immer mehr auf das häusliche Umfeld beschränkt.

## Prüfungsfragen

- Welche weiteren Fragen bzw. welche klinischen Untersuchungen interessieren Sie besonders?
- Welche weiterführende Diagnostik halten Sie für notwendig? Welche möglichen Differenzialdiagnosen bzw. Abgrenzungskriterien zwischen den Erkrankungen kommen in Betracht?
- Was wissen Sie über das Krankheitsbild hinsichtlich Entstehungsmechanismus und Diagnosekriterien?
- Was ist das bedeutendste Klassifikationssystem in der MRT?
- Wie kann die Prognose der Erkrankung abgeschätzt werden?
- Beschreiben Sie die Indikationen bzw. Verfahren der konservativen und operativen Behandlung?
- Welches weitere Vorgehen besprechen Sie mit Herrn H.?

### Antworten

#### Welche weiteren Fragen bzw. welche klinischen Untersuchungen interessieren Sie besonders?

##### Anamnese.

Seit wann bestehen die Schmerzen, sind sie nur bei Belastung/Gehen oder auch beim längeren Stehen vorhanden? Tritt eine Besserung nach einer Pause ein? Kamen die Schmerzen schleichend? Besteht ein inkliniertes Gangbild? Wird über eine Gefühlsstörung, kalte Füße oder Missempfindungen in den Beinen, z. B. Brennen, Ameisenlaufen, Kältegefühl, Wattegefühl unter den Füßen geklagt? Besteht bereits eine Schwäche der Beinmuskulatur? Besteht aktuell ein Hinweis für ein infektiöses Geschehen (Fieber, Krankheitsgefühl)? Voroperationen? Bestehen irgendwelche Vorerkrankungen (z. B. Stoffwechselerkrankungen/Gefäßprobleme)? Wenn ja, wurde bereits ein Arzt diesbezüglich konsultiert?

##### Der Fall.

Die Schmerzen werden im Bereich des unteren Rückens und vom Gesäß im Stehen und Gehen mit Ausstrahlung in die Beine bds. diffus angegeben (Müdigkeitsgefühl/„schwere“ Beine). Eine Pause mit Sitzgelegenheit ist unabdingbar zur Erholung. Die Gehstrecke ist auf 200–400 m reduziert. Ein auslösendes Trauma ist nicht erinnerlich, es bestehen internistische Vorerkrankungen: Diabetes mellitus Typ II, Adipositas, arterielle Hypertonie, koronare Herzerkrankung und initiale Polyneuropathie. Eine Infektion liege derzeit nicht vor. Eine Wirbelsäulenoperation hat noch nie stattgefunden.

##### Klinische Untersuchung.

Gezielt sollte nach spezifischen Befunden gesucht werden.

- Untersuchungsgang: Inspektion/Palpation/Funktionsprüfung am entkleideten Patienten
- Zeigt sich eine palpable Raumforderung?
- Besteht eine Einschränkung der Beweglichkeit der Hüft- und/oder der Kniegelenke?
- Lasègue-Test, Facettendruckschmerz, Rekliniations- und Inklinationsbeweglichkeit
- Kraftgarde der unteren Extremitäten nach Janda
- Hilfsmittel zum Gehen benötigt?
- Fußpulse (entscheidend)

##### Der Fall.

Es können an den warmen Füßen bds. kräftige Fußpulse getastet werden. Leicht inkliniertes Gangbild ohne Anzeichen eines Hinkens. Hüfte und Kniegelenke frei beweglich. Strumpfförmige Missempfindungen am rechten Fuß. Reklinationsschmerz im unteren Rücken mit Muskelhartspann. ISG (Iliosakralgelenk) bds. frei, kein Vorlaufphänomen. Eine Stresstestung mediolateral bzw. a.-p. zeigt keine Hinweise auf einen Bandscheibenschaden. Kein motorisches Defizit, Reflexe normgerecht seitengleich. Radfahren gehe problemlos. Inklination bringe Erleichterung.

#### Welche weiterführende Diagnostik halten Sie für notwendig? Welche möglichen Differenzialdiagnosen bzw. Abgrenzungskriterien zwischen den Erkrankungen kommen in Betracht?

##### Diagnostik.

- Röntgen der LWS (Lendenwirbelsäule) in 2 Ebenen mit Funktionsaufnahmen bei V. a. auf Spondylolisthesis
- MRT der LWS (CT nur bei Kontraindikationen zur MRT-Diagnostik oder wenn MRT nicht verfügbar im Umfeld)
- neurologische Untersuchung mittels EMG/NLG (Elektromyographie/Nervenleitgeschwindigkeit) zum Ausschluss einer Progredienz der Polyneuropathie

##### Differenzialdiagnosen.

[1]

- periphere arterielle Verschlusskrankheit (pAVK) der Gefäße
- raumeinengende Prozesse (Tumore, Tumormetastasen, Bandscheibenvorfall, Bandscheibenprotrusion) bei fehlender anatomischer (knöcherner) Enge oder traumatische und osteoporotische Veränderungen
- pseudoradikuläre Syndrome, Affektionen der ISG
- pelvine und inguinale Angiopathien
- Aortenaneurysma

##### Der Fall.

**Figure 1:**
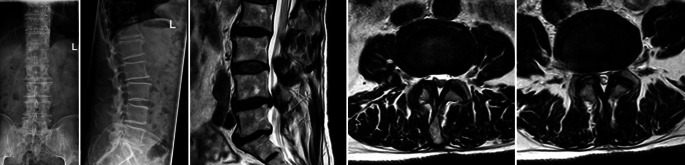

Aufgrund der tastbaren Fußpulse und warmen Füße ist eine pAVK unwahrscheinlich. Anhand der klinischen Untersuchung zeigt sich in diesem Fall keinerlei myofasziale Dysfunktion sowie eine pseudoradikuläre Symptomatik. In der MRT der Lendenwirbelsäule können eine Metastase oder ein Bandscheibenvorfall ausgeschlossen werden, und es ergibt sich die Diagnose einer Spinalkanalstenose (Abb. 1).

#### Was wissen Sie über das Krankheitsbild hinsichtlich Entstehungsmechanismus und Diagnosekriterien?

##### Krankheitsbild.

Es ist ein Erkrankungsbild des höheren Lebensalters in dem der Wirbelkanal, hier lumbal, durch Kompression/Einengung der Nerven und Gefäße Beschwerden verursacht. Die Ursachen sind in vielen Fällen eine Kombination aus mehreren Faktoren und können angeboren oder erworben sein:

- Die degenerativen Veränderungen an den kleinen Wirbelgelenken (Spondylarthrose), Hypertrophie des Lig. flavum, die Ausbildung juxtaartikulärer Zysten, Bandscheibenvorfälle und Tumoren können zu einer Spinalkanalstenose führen oder Nervenwurzeln im Recessus lateralis oder Neuroforamen bedrängen.
- Bei jungen Patienten besteht meistens ein kongenital eng angelegter Spinalkanal. Hier ist aufgrund der kurzen Pedikel der Durchmesser des Spinalkanales deutlich eingeschränkt im Vergleich zu den Normwerten.

#### Was ist das bedeutendste Klassifikationssystem in der MRT?

**Figure 2:**
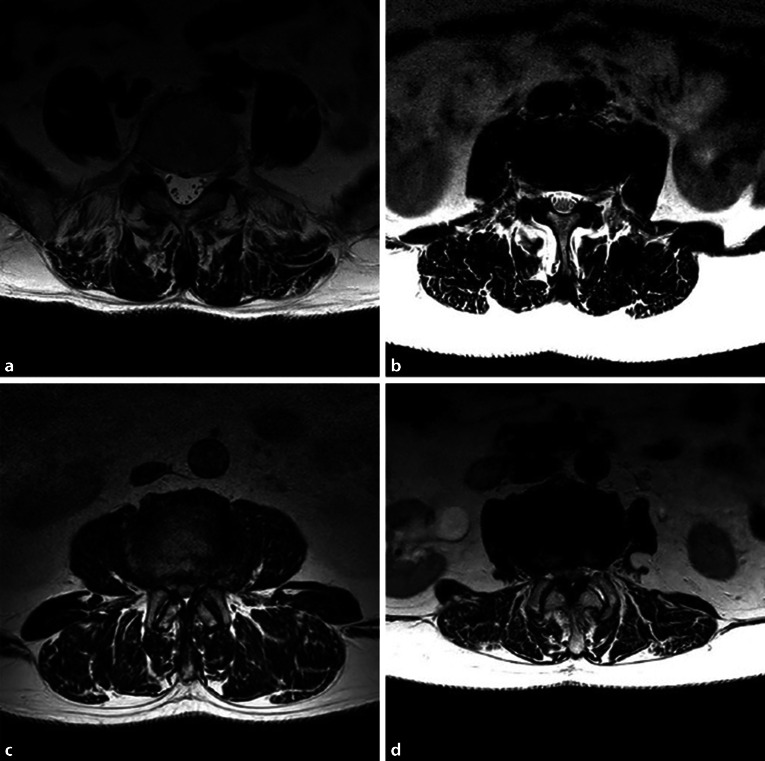

Magnetresonanztomographie-basierte **Einteilung der lumbalen Spinalkanalstenose nach Schizas** [2]:

- **Typ A**: Die Nervenfasern besetzen weniger als 50 % des Durasacks und sind von Liquor umgeben.
- **Typ B**: Die Nervenfasern füllen den ganzen Duraraum aus, aber können noch voneinander unterschieden werden. Etwas Liquor ist noch sichtbar und gibt dem Durasack ein körniges Aussehen.
- **Typ C**: Die Nervenfasern sind nicht sichtbar, der Duraraum stellt sich homogen grau ohne Liquorsignal dar. Posterior zeigt sich noch epidurales Fett.
- **Typ D**: Zu den nicht erkennbaren Nervenfasern von Typ C kommt auch noch die Abwesenheit von epiduralem Fett (Abb. 2).

#### Wie kann die Prognose der Erkrankung abgeschätzt werden?

Die Prognose der Erkrankung besteht in einer langsamen Zunahme der Beschwerden mit zwischenzeitlichen Besserungstendenzen. Die Gehstrecke wird sich im weiteren zeitlichen Verlauf vermindern. Es entsteht ein inkliniertes Gangbild. Am Ende des Therapieschemas steht die operative Versorgung zur Erweiterung des Spinalkanales, beziehungsweise zur Dekompression der Nervenwurzeln. Anfänglich ist sicherlich eine konservative Therapie den Beschwerden und der Neurologie (Blasen‑, Mastdarmschwäche, gestörte Sexualfunktion) entsprechend indiziert.

##### Der Fall.

Bei Herrn H. zeigt sich ein leicht inkliniertes Gangbild, die Gehstrecke ist noch nicht stark eingeschränkt, schließlich müsse er mit dem Hund täglich raus, weshalb er das noch gut abschätzen könne. Dies sei sein Training. An schlechten Tagen nehme er Schmerztabletten. Die Gehstrecke ist bei der klinischen Untersuchung bis zum Stehen bleiben 350 m, dann tritt ein hinkendes Gangbild mit einem Schweregefühl der Beine auf.

#### Beschreiben Sie die Indikationen bzw. Verfahren der konservativen/operativen Behandlung?

##### Konservative Therapie.

- **Indikation**: bei fehlender gravierender Neurologie/Paresen/Konus-Kauda-Symptomatik
- **Techniken**: Kombination aus Physiotherapie mit Rückenschule und Wärme‑/Kältetherapien zur Anregung der Durchblutung/Entlastung der Muskulatur, Elektrotherapie, Stützkorsett, Schmerztherapie mit Erlernen von Schmerzbewältigungskonzepten, selektive Infiltrationen zur Symptomlinderungen, Gang‑, Standschule, Vitamin-D-Substitution zur Verbesserung der Muskelstruktur und Muskelkraft [4]

##### Operative Therapie.

**Figure 3:**
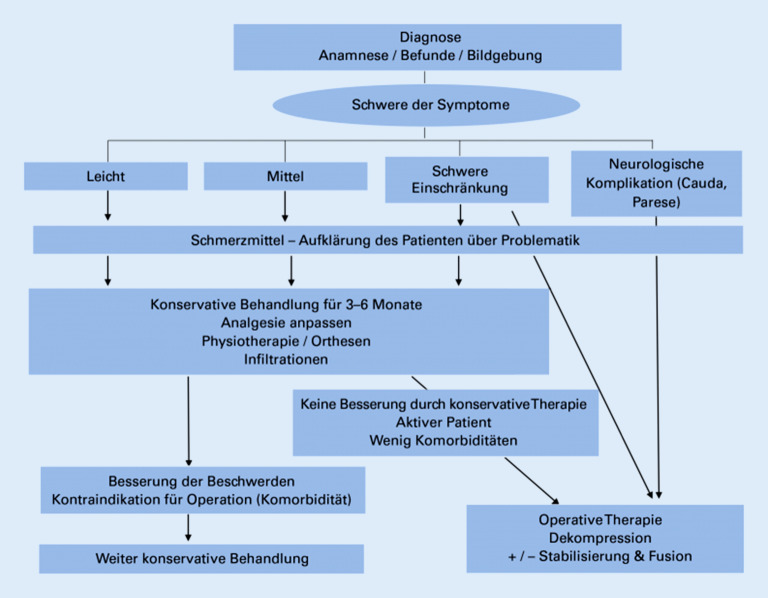

- **Indikation**: Versagen der konservativen Therapie, „relevante“ neurologische Ausfälle
- **Techniken**: Dekompression der neuralen Strukturen mittels mikrochirurgischer Dekompression ohne und mit Laminektomie, sowie Fusion des Segmentes, Fusionsoperation bei Spondylolisthesis, minimal-invasive mikrochirurgische oder endoskopische Dekompression mittels Laminotomie und Rezessusdekompression [5, 6]
- Bei allen operativen Versorgungen besteht die Gefahr einer Verletzung der Dura mit Liqourleckage (Abb. 3).

##### Der Fall.

Bei Herrn H. wird die konservative Therapie indiziert. Es zeigt sich im Verlauf über 6 Monte initial eine leichte Besserung, schlussendlich jedoch kein durchgreifender Therapieerfolg. Der Patient ist damit unzufrieden, die Gehstrecke ist eher kürzer geworden. Eine relevante pAVK konnte zwischenzeitlich ausgeschlossen werden.

#### Welches weitere Vorgehen besprechen Sie mit Herrn H.?

- Da sich Herr H. bereits seit längeren in konservativer Therapie mittels Physiotherapie, oraler Schmerztherapie und durch selektive Bildwandler gestützte Infiltrationen befindet, die konservative Therapie keinen Erfolgt mehr erzielt und die Differenzialdiagnose pAVK (bei starken Rauchern und Diabetes mellitus Typ II) ausgeschlossen werden konnte, kann man nun über eine operative Maßnahme mit dem Patienten diskutieren.
- Ziel der Operation ist eine Erweiterung des Spinalkanals und Dekompression der neuralen Strukturen. Diese Operation ist aufgrund fehlender Instabilität in dem Segment minimal-invasiv durchführbar. Ob dies nun durch eine endoskopische oder mikrochirurgische Dekompression erfolgt, ist dem Operateur überlassen. Beide Verfahren sind als gleichwertig zu betrachten.
